# Activation of Free Fatty Acid Receptor 4 (FFA4) Ameliorates Ovalbumin-Induced Allergic Asthma by Suppressing Activation of Dendritic and Mast Cells in Mice

**DOI:** 10.3390/ijms23095270

**Published:** 2022-05-09

**Authors:** So-Eun Son, Jung-Min Koh, Dong-Soon Im

**Affiliations:** 1 Department of Biomedical and Pharmaceutical Sciences, Graduate School, Kyung Hee University, Seoul 02447, Korea; seson@khu.ac.kr; 2 Division of Endocrinology and Metabolism, Asan Medical Center, University of Ulsan College of Medicine, Seoul 05505, Korea; jmkoh@amc.seoul.kr; 3 Department of Basic Pharmaceutical Sciences, Graduate School, Kyung Hee University, Seoul 02447, Korea

**Keywords:** allergy, asthma, free fatty acid receptor 4, FFA4, omega-3 polyunsaturated fatty acids

## Abstract

Epidemiological and clinical studies have suggested that intake of n-3 polyunsaturated fatty acids (PUFA) reduces the incidence of allergic airway diseases and improves pulmonary function in patients with allergic asthma. However, the pharmacological targets of PUFA have not been elucidated upon. We investigated whether free fatty acid receptor 4 (FFA4, also known as GPR120) is a molecular target for beneficial PUFA in asthma therapy. In an ovalbumin (OVA)-induced allergic asthma model, compound A (a selective agonist of FFA4) was administrated before OVA sensitization or OVA challenge in FFA4 wild-type (WT) and knock-out (KO) mice. Compound A treatment of RBL-2H3 cells suppressed mast cell degranulation in vitro in a concentration-dependent manner. Administration of compound A suppressed in vivo allergic characteristics in bronchoalveolar lavage fluid (BALF) and lungs, such as inflammatory cytokine levels and eosinophil accumulation in BALF, inflammation and mucin secretion in the lungs. Compound A-induced suppression was not only observed in mice treated with compound A before OVA challenge, but in mice treated before OVA sensitization as well, implying that compound A acts on mast cells as well as dendritic cells. Furthermore, this suppression by compound A was only observed in FFA4-WT mice and was absent in FFA4-KO mice, implying that compound A action is mediated through FFA4. Activation of FFA4 may be a therapeutic target of PUFA in allergic asthma by suppressing the activation of dendritic cells and mast cells, suggesting that highly potent specific agonists of FFA4 could be a novel therapy for allergic asthma.

## 1. Introduction

Asthma—an inflammatory airway disease—is mainly driven by immune responses triggered by increased airway eosinophils and T helper 2 (T_H_2) cells, mucus hypersecretion, and secretion of pro-inflammatory cytokines [1]. Inhaled glucocorticoids and antileukotriene drugs have been used for asthma therapy [2]; however, non-responding patients with moderate-to-severe asthma require safe alternative therapeutic options for asthma management. Epidemiologically, the prevalence of asthma, coronary heart disease, rheumatoid arthritis, and psoriasis was historically low among Inuit, and n-3 polyunsaturated fatty acids (PUFA)—such as docosahexaenoic acid (DHA) and eicosapentaenoic acid (EPA)—found in dietary oily fish have been suggested to be a cause for the lack of prevalence of such conditions [3]. Dietary supplementation with n-3 PUFA in asthmatic patients could reduce bronchial inflammation [4,5]. Dietary supplementation with n-3 PUFA in children with bronchial asthma has, for example, been found to be beneficial in terms of inhalant allergens [6]. However, dietary fish oil supplementation in pollen-sensitive subjects did not prevent seasonal hay fever or asthma during the pollen season [7]. Furthermore, a review suggested the health effects of n-3 PUFA in asthma- and exercise-induced bronchoconstriction [8]. However, a major obstacle in n-3 PUFA studies has been the lack of specific targets at the molecular level.

The modes of action of n-3 PUFA on asthma symptoms have been extensively studied for a significant period of time. Two modes of action have been suggested as: (1) competition between n-3 PUFA and arachidonic acid in the production of pro-inflammatory eicosanoids; and (2) the conversion of n-3 PUFA to specialized pro-resolving lipid mediators, such as resolvins [9]. However, a possibility has not been studied: the possibility that free fatty acid receptor 4 (FFA4, also known as GPR120), a specific n-3 PUFA sensor, may act as a molecular target of the beneficial n-3 PUFA effects on allergic asthma [10,11]. FFA4 has been found to be expressed in the lungs and immune cells, including monocytes, macrophages, dendritic cells, and eosinophils [12,13,14,15,16]. The anti-inflammatory functions of FFA4 were also reported to be mediated through M2 macrophage polarization in vivo [11,17]. Therefore, we used an ovalbumin (OVA)-induced allergic asthma model to investigate whether FFA4 is a molecular target of n-3 PUFA in asthma therapy in FFA4 gene wild-type (WT) and knock-out (KO) mice in combination with compound A, a selective agonist of FFA4 [17,18].

## 2. Results

### 2.1. Compound A Suppressed Degranulation of Mast Cells

**Figure 1:**
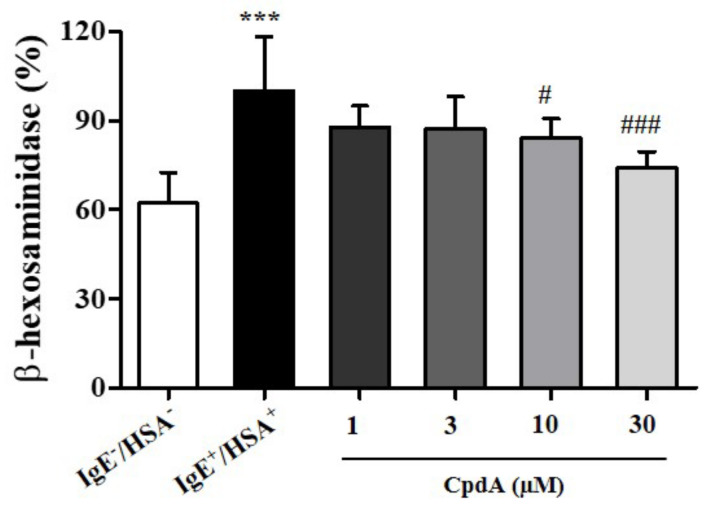
Compound A suppresses antigen-induced degranulation in RBL-2H3 cells. RBL-2H3 cells were sensitized with anti-DNP IgE for 18 h and challenged with DNP human serum albumin (HSA). Compound A was added at the indicated concentrations 30 min before HSA challenge. Samples without IgE and HSA showed basal degranulation, and samples with IgE and HSA showed positive control of antigen-induced degranulation. The results are presented as mean ± standard error (SE) of five independent experiments. *** *p* < 0.001 vs. the HSA-untreated group. # *p* < 0.05, ### *p* < 0.001 vs. the HSA-treated group.

Mast cells are tissue-resident hematopoietic cells that contribute to adaptive immune responses by binding antibodies through FcεRI receptors in allergies and asthma [19]. Antigen-induced cross-linking of FcεRI on mast cell membranes results in degranulation [20]. Degranulation releases a plethora of potent immune mediators, such as histamine, leukotrienes, and prostaglandins [21]. Recently, n-3 PUFA were shown to inhibit FcεRI localization and signaling in the LAD2 human mast cell line [22]. FFA4 expression in mast cells and its involvement in the inhibitory effect of n-3 PUFA on degranulation have been implicated [22]. First, we tested the effects of compound A on degranulation of rat RBL-2H3 mast cells by measuring β-hexosaminidase activity in the medium (Figure 1). Treatment of compound A suppressed the release of β-hexosaminidase in a concentration-dependent manner (Figure 1), confirming previous observations in human mast cells [22]. The inhibition was significant at concentrations of 10 and 30 µM of compound A (Figure 1).

### 2.2. Compound A Ameliorated Asthma-Induced Influx of Immune Cells in FFA4-WT Mice

**Figure 2:**
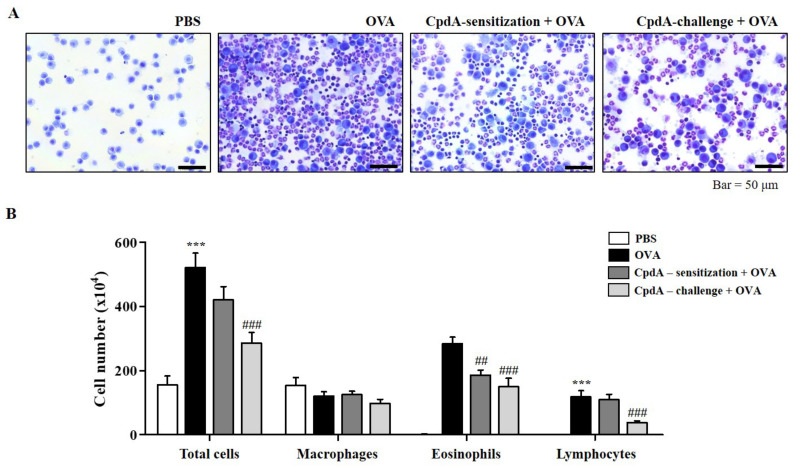
Compound A suppresses OVA-induced immune cell accumulation in BALF of FFA4-WT mice. (**A**) Mice were sensitized with OVA twice by i.p. injection on day 0 (D0) and D14, and later challenged on D28, D29, and D30 with nebulized OVA. Compound A was administrated intraperitoneally at the dose of 30 mg/kg, 30 min before OVA sensitization or before OVA challenge. BALF cells were stained using May-Grünwald stain and counted. (**B**) Total cell counts, eosinophils, macrophages, and lymphocytes in BALF. The results are presented as the mean ± SEM of cell count values (*n* = 5). *** *p* < 0.001 vs. the PBS-treated group, ## *p* < 0.01, ### *p* < 0.001 vs. the OVA-treated group.

The in vitro suppressive effect of compound A on the degranulation of RBL-2H3 cells led to the establishment of an in vivo experiment on OVA-induced allergic asthma in FFA4-WT mice. Experimental groups were designed for treating with compound A not only before OVA challenge, but also before OVA sensitization. After the induction of allergic asthma, many immune cells were collected from BALF (Figure 2A). In the BALF, the total cell number increased to 253.6% in the OVA-induced asthma group, and both treatments of compound A before challenge and before sensitization reduced the total cell numbers (Figure 2B). However, a significant difference of 67.4% was observed, but only in compound A treatment before challenge (Figure 2B). Eosinophils were collected by OVA treatment; significantly less were collected in BALF by both compound A treatments (Figure 2B). Treatment with compound A before the challenge also significantly suppressed the number of lymphocytes (Figure 2B). Treatment with OVA or compound A did not significantly change the number of macrophages in this model (Figure 2B).

### 2.3. Compound A Ameliorated Asthma-Induced Inflammation and Mucin Secretion in the Lungs of FFA4-WT Mice

**Figure 3:**
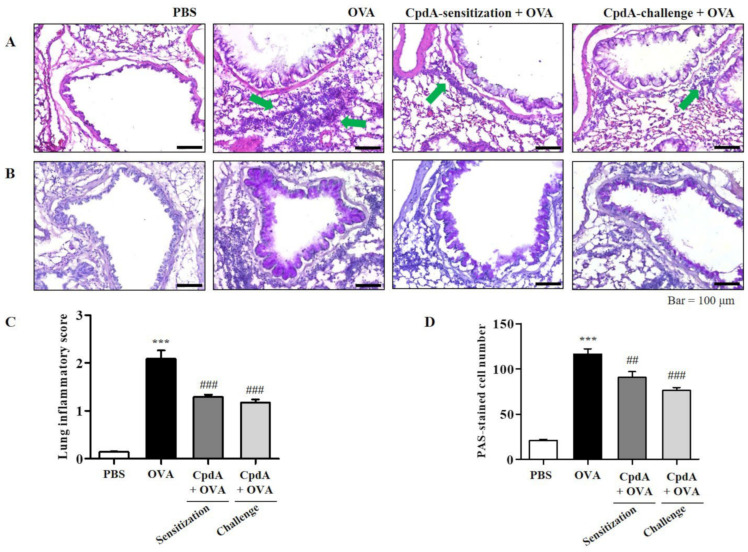
Compound A protects against airway inflammation and mucin secretion in FFA4-WT mice. (**A**) Panels show H&E-stained sections of lung tissues from the PBS group, OVA group, and compound A-treated OVA groups (before sensitization or before challenge). Small navy-blue dots around the bronchioles indicate eosinophils. Eosinophils are accumulated around bronchioles in the OVA group (green arrows). (**B**) Panels show PAS/hematoxylin-stained sections of lung tissues from the PBS group, OVA group, and compound A-treated OVA groups (before sensitization or before challenge). In PAS staining, mucin is stained as a purple color. Darker and thicker purple coloring is observed surrounding the bronchiole in the OVA group compared to the PBS group. (**C**) Lung inflammation was semi-quantitatively evaluated; histological findings were scored as described in Section 4. (**D**) Mucous production was measured by counting the number of PAS-positive cells per mm of bronchiole (*n* = 5 per group). Values represent the means ± SEM (*n* = 5). *** *p* < 0.001 vs. the PBS-treated group, ## *p* < 0.01, ### *p* < 0.001 vs. the OVA-treated group.

The degree of inflammation and mucin secretion was estimated by inspecting lung tissues. The influx of eosinophils in the lung tissues was confirmed visually by H&E staining; they surrounded the airways, indicated by green arrows in the OVA group (Figure 3A). Eosinophil accumulation was lower in the compound A-treated groups (Figure 3A). Mucins were visualized by PAS staining. Violet-colored mucins were rare in the PBS-treated group, but strong PAS staining was observed around the bronchioles in the OVA group (Figure 3B). In the compound A-treated groups, the staining was weaker than that of the OVA group, suggesting suppression of mucin production (Figure 3B).

The degrees of lung inflammation and mucin production were analyzed using a subjective scale of 0–3, semi-quantitative evaluation of lung inflammation, and via the counting of PAS-positive cells in bronchioles as previously described [20,22]. The mean inflammation score in the OVA-treated group was about 2.1, and treatment with compound A reduced the inflammation score to approximately 1.2 (Figure 3C). In the OVA-treated group, approximately 110 PAS-positive cells/mm were detected, and both treatments with compound A significantly suppressed the number of PAS-positive cells (Figure 3D). Therefore, OVA-induced increases in eosinophil and mucin levels in the lungs were significantly suppressed via treatment with compound A in FFA4-WT mice (Figure 3).

### 2.4. Compound A Ameliorated Asthma-Induced Cytokine Expression in FFA4-WT Mice

**Figure 4:**
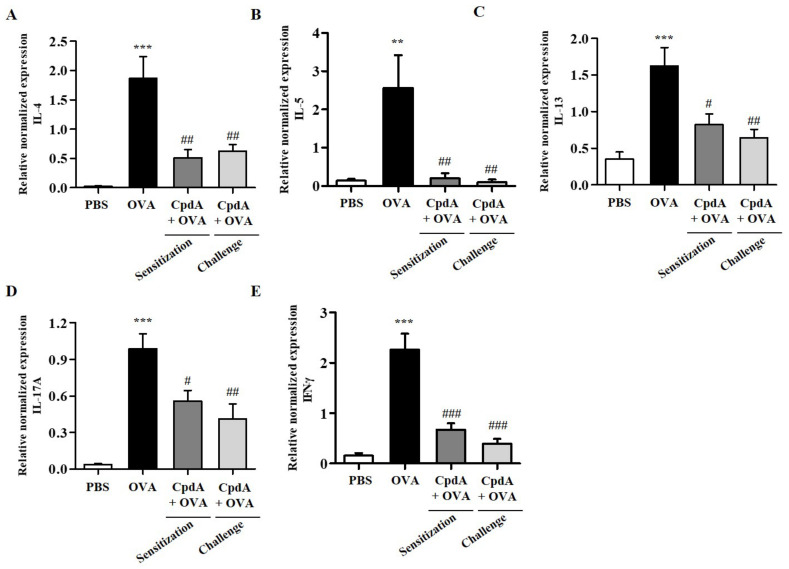
Effect of compound A on expression levels of T_H_2, T_H_17, and T_H_1 cytokines in the BALF of OVA-induced asthma in FFA4-WT mice. Analysis of mRNA expression of T_H_2 (IL-4, IL-5, and IL-13), T_H_17 (IL-17A), and T_H_1 (IFN-γ) cytokines in BALF of OVA-induced and compound A-treated FFA4-WT mice. Relative mRNA levels of cytokines were quantified as ratios to GAPDH transcript levels. (**A**) IL-4, (**B**) IL-5, (**C**) IL-13, (**D**) IL-17A, and (**E**) IFN-γ. The results are presented as mean ± SEM (*n* = 5). ** *p* < 0.01, *** *p* < 0.001 vs. the PBS-treated group, # *p* < 0.05, ## *p* < 0.01, ### *p* < 0.001 vs. the OVA-treated group.

Upon recognizing allergens, allergen-specific T_H_2 cells produce type 2 cytokines (IL-4, IL-5, and IL-13), which leads to the accumulation of high numbers of eosinophils in the airway wall, mucus overproduction, and synthesis of immunoglobulin E (IgE) [23]. Furthermore, T_H_1 and T_H_17 cytokines are suggested to be involved in the pathogenesis of asthma in the late stage of chronic asthma [24]. Therefore, changes in the mRNA levels of T_H_2/T_H_1/T_H_17 cytokines, IL-4, IL-5, IL-13, IFN-γ, and IL-17A were assessed in BALF immune cells by qPCR. As shown in Figure 4, the mRNA levels of all five cytokines were increased in BALF cells from the OVA group, and both treatments with compound A suppressed all five cytokines (Figure 4). 

**Figure 5:**
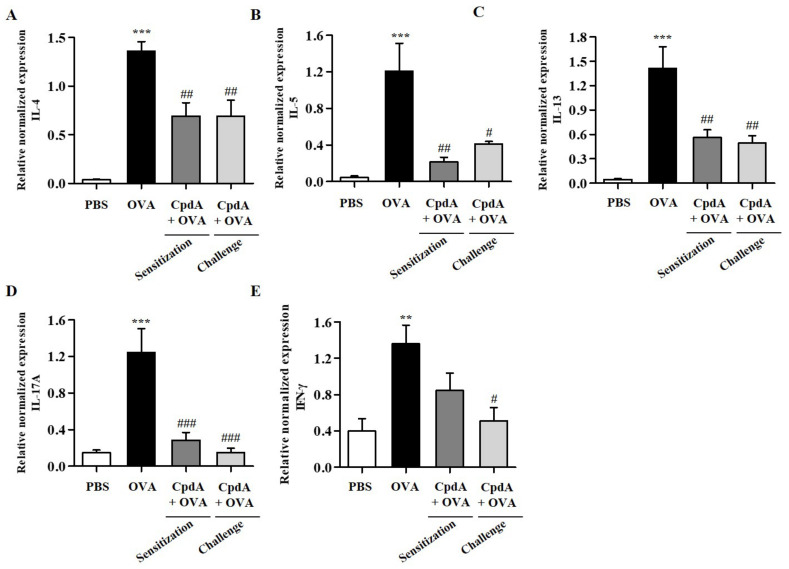
Effect of compound A on expression levels of T_H_2, T_H_17, and T_H_1 cytokines in the lung tissues of OVA-induced asthma in FFA4-WT mice. Analysis of mRNA expression of T_H_2 (IL-4, IL-5, and IL-13), T_H_17 (IL-17A), and T_H_1 (IFN-γ) cytokines in the lung tissues of OVA-induced and compound A-treated FFA4-WT mice. Relative mRNA levels of cytokines were quantified as ratios to GAPDH transcript levels. (**A**) IL-4, (**B**) IL-5, (**C**) IL-13, (**D**) IL-17A, and (**E**) IFN-γ. The results are presented as mean ± SEM (*n* = 5). ** *p* < 0.01, *** *p* < 0.001 vs. the PBS-treated group, # *p* < 0.05, ## *p* < 0.01, ### *p* < 0.001 vs. the OVA-treated group.

The mRNA levels of T_H_2/T_H_1/T_H_17 cytokines were determined in the lungs. As shown in Figure 5, the mRNA levels of the five cytokines were significantly increased in the lungs of mice in the OVA group, and both treatments with compound A suppressed the five cytokines (Figure 5).

### 2.5. Compound A Did Not Ameliorate Asthma-Induced Influx of Immune Cells in FFA4-KO Mice

**Figure 6:**
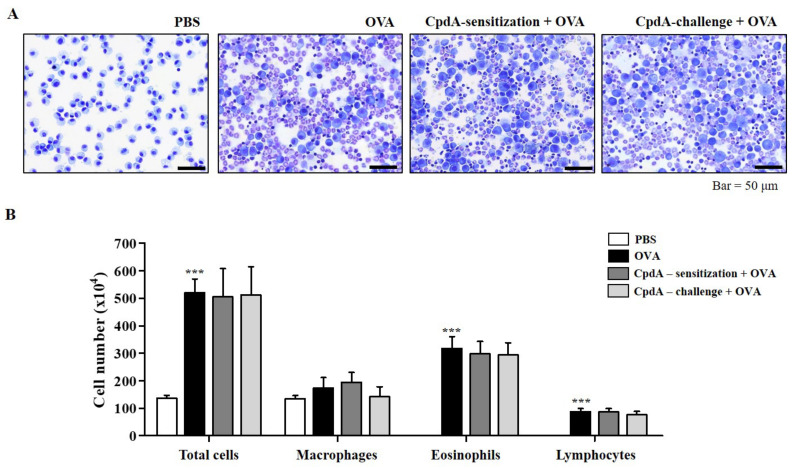
Compound A does not suppress OVA-induced immune cell accumulation in BALF of FFA4-KO mice. (**A**) Mice were sensitized and challenged, and compound A was administrated as described in FFA4-WT mice. BALF cells were stained using May-Grünwald stain and counted. (**B**) Total cell counts, eosinophils, macrophages, and lymphocytes in BALF. The results are presented as the mean ± SEM of cell count values (*n* = 5). *** *p* < 0.001 vs. the PBS-treated group.

Next, we verify whether the effect of compound A was mediated through FFA4 in FFA4-KO mice. Compound A treatment did not suppress eosinophil influx into the BALF of the FFA4-KO mice (Figure 6A). In FFA4-KO mice, the total cell number increased to 281.9% in the OVA-induced asthma group, but neither treatment with compound A changed the OVA-induced increase in the total cell number (Figure 6B). In addition, the increased numbers of eosinophils and lymphocytes induced by OVA were not suppressed by treatment with compound A in FFA4-KO mice (Figure 6B). Macrophage numbers remained unchanged by OVA and compound A in mice (Figure 6B).

### 2.6. Compound A Did Not Ameliorate Asthma-Induced Inflammation and Mucin Secretion in the Lungs of FFA4-KO Mice

**Figure 7:**
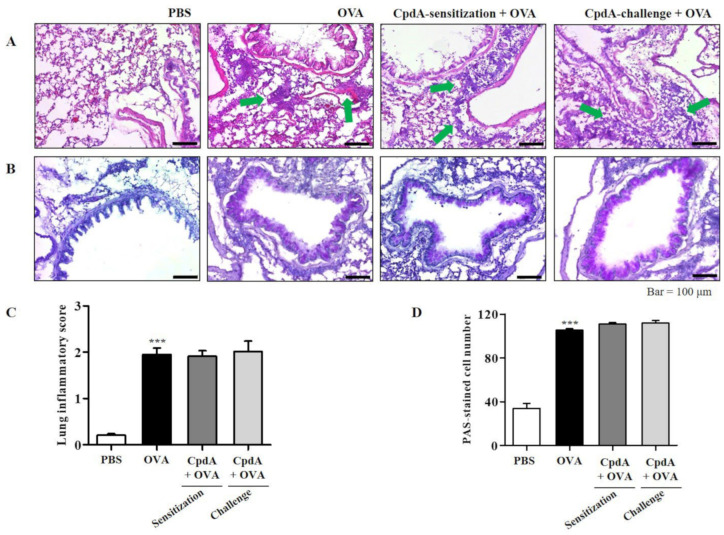
Compound A does not protect against airway inflammation and mucin secretion in FFA4-KO mice. (**A**) Panels show H&E-stained sections of lung tissues from the PBS group, OVA group, and compound A-treated OVA groups (before sensitization or before challenge). Small dark-blue dots around the bronchioles indicate eosinophils. Eosinophils are accumulated around bronchioles in the OVA group (green arrows). (**B**) Panels show PAS/hematoxylin-stained sections of lung tissues from the PBS group, OVA group, and compound A-treated OVA groups (before sensitization or before challenge). In PAS staining, mucin is stained as a purple color. Darker and thicker purple coloring is observed surrounding the bronchiole in the OVA group compared to the PBS group. (**C**) Lung inflammation was semi-quantitatively evaluated; histological findings were scored as described in Section 4. (**D**) Mucous production was measured by counting the number of PAS-positive cells per mm of bronchiole (*n* = 5 per group). Values represent the mean ± SEM (*n* = 5). *** *p* < 0.001 vs. the PBS-treated group.

Similar to FFA4-WT mice, H&E staining of the lungs confirmed the influx of eosinophils surrounding the airways in the OVA group; however, eosinophil accumulation was not suppressed by treatment with compound A in FFA4-KO mice (Figure 7A). Degrees of lung inflammation were also analyzed, and the mean inflammation score was 2.0 in the OVA-treated group, but both treatments with compound A did not suppress the inflammation score (Figure 7C). 

PAS staining showed a violet-colored surface around the bronchioles in the OVA group of FFA4-KO mice, similar to what had been observed in FFA4-WT mice (Figure 7B). However, the staining was not suppressed by treatment with compound A in FFA4-KO mice (Figure 7B). Semi-quantitative measurement of mucins showed approximately 100 PAS-positive cells/mm in the OVA-treated group of FFA4-KO mice, and neither treatment with compound A suppressed the numbers of PAS-positive cells (Figure 7D). In summary, increased inflammation and mucin hyperproduction in the lungs of OVA-treated asthmatic mice were maintained in FFA4-KO mice, and neither treatment with compound A suppressed them (Figure 7). 

### 2.7. Compound A Did Not Ameliorate Asthma-Induced Cytokine Expression in FFA4-KO Mice

**Figure 8:**
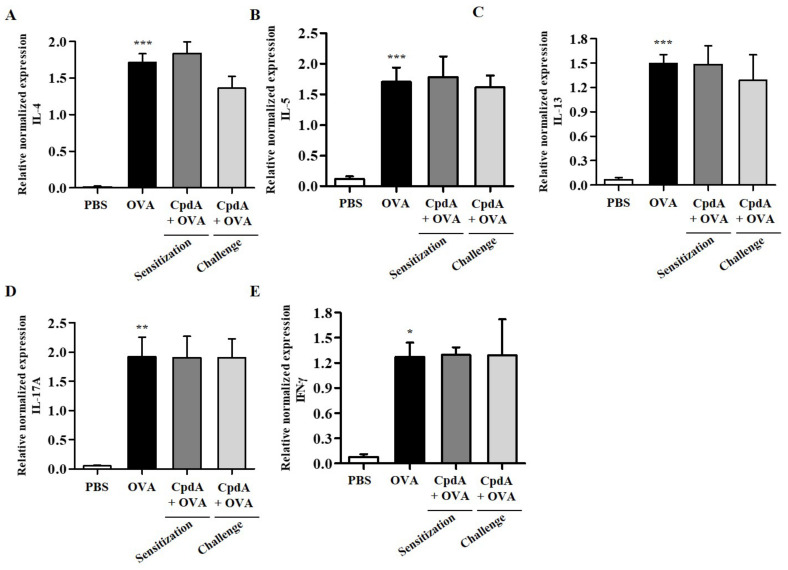
Effect of compound A on expression levels of T_H_2, T_H_17, and T_H_1 cytokines in the BALF of OVA-induced asthma in FFA4-KO mice. Analysis of mRNA expression of T_H_2 (IL-4, IL-5, and IL-13), T_H_17 (IL-17A), and T_H_1 (IFN-γ) cytokines in BALF of OVA-induced and compound A-treated FFA4-KO mice. Relative mRNA levels of cytokines were quantified as ratios to GAPDH transcript levels. (**A**) IL-4, (**B**) IL-5, (**C**) IL-13, (**D**) IL-17A, and (**E**) IFN-γ. The results are presented as mean ± SEM (*n* = 5). * *p* < 0.05, ** *p* < 0.01, *** *p* < 0.001 vs. the PBS-treated group.

As in FFA4-WT mice, T_H_2/T_H_1/T_H_17 cytokine levels were determined in BALF immune cells using qPCR. As shown in Figure 8, the mRNA levels of all five cytokines were increased in the BALF cells of the OVA group in FFA4-KO mice (Figure 8). However, these increases were not suppressed via treatment with compound A in FFA4-KO mice (Figure 8). 

**Figure 9:**
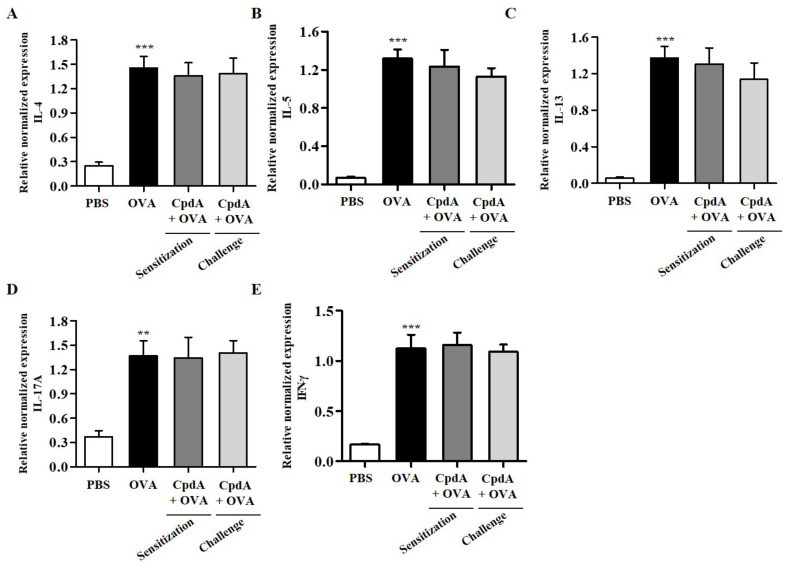
Effect of compound A on expression levels of T_H_2, T_H_17, and T_H_1 cytokines in the lung tissues of OVA-induced asthma in FFA4-KO mice. Analysis of mRNA expression of T_H_2 (IL-4, IL-5, and IL-13), T_H_17 (IL-17A), and T_H_1 (IFN-γ) cytokines in lung tissues of OVA-induced and compound A-treated FFA4-KO mice. Relative mRNA levels of cytokines were quantified as ratios to GAPDH transcript levels. (**A**) IL-4, (**B**) IL-5, (**C**) IL-13, (**D**) IL-17A, and (**E**) IFN-γ. The results are presented as mean ± SEM (*n* = 5). ** *p* < 0.01, *** *p* < 0.001 vs. the PBS-treated group.

The expression levels of T_H_2/T_H_1/T_H_17 cytokines were also analyzed in the lungs of FFA4-KO mice. The mRNA levels of all five cytokines increased in the lung tissue of the OVA group in FFA4-KO mice (Figure 9). However, these increases were not inhibited by compound A in FFA4-KO mice (Figure 9).

### 2.8. Changes of Serum IgE and BALF Cytokine Levels by OVA and Compound A in Mice

**Figure 10:**
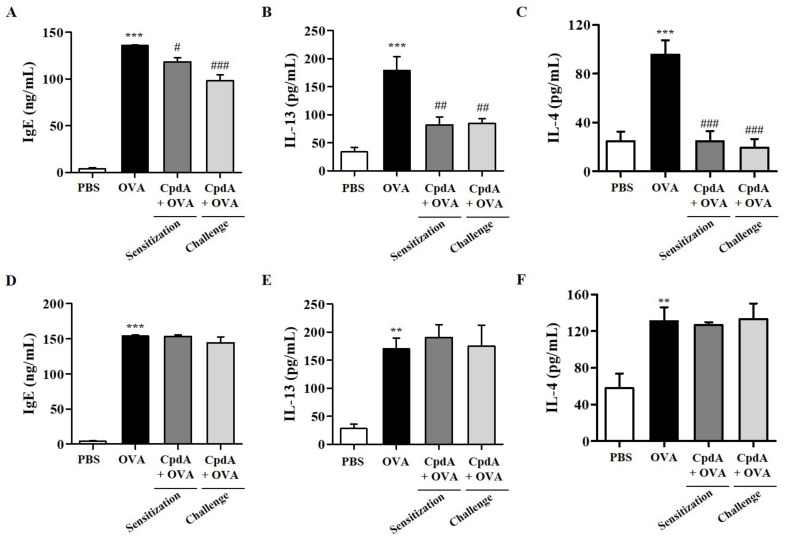
Effect of compound A on IgE levels in serum and levels of IL-13 and IL-4 cytokines in BALF. Serum IgE levels in FFA4-WT mice (**A**) and FFA4-KO mice (**D**). The results are presented as mean ± SEM (*n* = 5). *** *p* < 0.001 vs. the PBS-treated group, # *p* < 0.05, ### *p* < 0.001 vs. the OVA-treated group. ELISA was used to measure the protein levels of IL-13 and IL-4 in BALF from FFA4-WT mice (**B**,**C**) and FFA4-KO mice (**E**,**F**). The results represent the mean ± SEM of protein levels (*n* = 5). ** *p* < 0.01, *** *p* < 0.001 vs. the PBS-treated group. ## *p* < 0.01, ### *p* < 0.001 vs. the OVA-treated group.

Serum IgE levels were assessed to confirm the immunological effects of OVA and compound A in the FFA4-WT and FFA4-KO mice. IgE production increased in the sera of OVA-treated FFA4-WT and FFA4-KO mice (Figure 10A,D). An OVA-induced increase in serum IgE levels was partly suppressed via treatment with compound A in FFA4-WT mice, but not in FFA4-KO mice (Figure 10A,D).

The protein levels of T_H_2 cytokine IL-13 and IL-4 in BALF were measured by ELISA. IL-13 levels were significantly increased in the OVA-induced group in both FFA4-WT and FFA4-KO mice, and both treatments of compound A significantly suppressed IL-13 levels in FFA4-WT mice but not in FFA4-KO mice (Figure 10B,E). IL-4 levels were increased in the OVA-induced group of FFA4-WT mice, and both treatments with compound A demonstrated a statistically-significant suppression of IL-4 levels in FFA4-WT mice (Figure 10C). IL-4 levels were increased by OVA treatment but were not suppressed by treatment with compound A in FFA4-KO mice (Figure 10F).

## 3. Discussion

Epidemiological, preclinical, and clinical studies have supported the beneficial effects of n-3 PUFA on airway inflammation and allergic asthma [3,4,5,6,7,8]. In addition, downregulation of n-3 PUFA levels in serum accompanied T_H_2-mediated inflammation, implying that n-3 PUFA supplementation affects T_H_2 immune responses [25]. There are two mechanisms underlying the beneficial effects of n-3 PUFA on asthma and airway inflammation. 

The first is the competition between n-3 PUFA with arachidonic acid for cyclooxygenase and lipoxygenase in the production of eicosanoids [9]. This could result in decreased production of pro-inflammatory cytokines, such as prostaglandin E_2_ and leukotriene D_4_, and a reduction of immune cell function [9]. Supplementation with EPA increased EPA content 10-fold, which was linked to the inhibition of pro-inflammatory leukotriene B_4_ generation in neutrophils [26]. Aerosolized DHA exposure reduced bronchial hyper-responsiveness and cell infiltration in a murine asthma model [27]. 

The second is the conversion of n-3 PUFA to specialized pro-resolving mediators such as resolvin E1 and resolvin D1 [28]. In fat-1 transgenic mice, elevated n-3 PUFA increased the production of protectin D1 and resolvin E1 and protected against allergic airway responses [20]. In practice, both protectin D1 and resolvin E1 can suppress allergic airway inflammation and hyper-responsiveness [29,30,31]. Mechanistically, resolvin E1 regulates IL-23, IL-6, IFN-γ, and lipoxin A_4_ to promote resolution of allergic airway inflammation [32]. 

In this study, we utilized compound A—a specific agonist of FFA4—in order to exclude these two mechanisms. We found that compound A administration suppressed allergic responses through FFA4, which was clearly demonstrated in FFA4-KO mice, strongly suggesting a third mode of action of n-3 PUFA in allergic asthma. Surprisingly, compound A treatment both before OVA challenge and before OVA sensitization equally protected against asthma development. The suppression by compound A treatment before the challenge could be explained by the suppression of mast cell activation, which was demonstrated in RBL-2H3 cells in the present study, as mast cells play a very important role in the period of allergen challenges. Previously, Wang et al. reported an inhibitory effect of n-3 PUFA on FcεRI localization and signaling in mouse bone marrow-derived mast cells [33]. Furthermore, the role of FFA4 receptors in mast cell degranulation has been demonstrated in human mast cells [22]. Therefore, the suppressive in vivo results of compound A treatment before OVA challenge may be mediated through suppression of mast cell activation by compound A in the antigen challenge.

Another observation was asthma suppression by compound A treatment before OVA sensitization. This suppression may be explained by the suppression of dendritic cell activation. Dendritic cells play a pivotal role in antigen sensitization by recognizing and presenting antigens through maturation process [34]. Previously, n-3 PUFA treatment was shown to affect human monocyte-derived dendritic cell differentiation and block dendritic cell activation by LPS, resulting in the suppression of surface markers, CD40, CD80, CD86, and MHC II, and suppression of TNF-α, IL-12p40, and COX-2 mRNA levels [35,36,37,38,39]. N-3 PUFA suppressed LPS-induced dendritic cell maturation through FFA4 [38,39,40]. The n-3 PUFA suppression of dendritic cell maturation resulted in an in vivo increase in CD4^+^Foxp3^+^ T regulatory cells and a decrease in CD4^+^IL-17^+^ T cells [38,39]. In an experimental autoimmune encephalomyelitis model, DHA ameliorated autoimmune inflammation by activating the FFA4 signaling pathway in dendritic cells, which was mediated mechanistically through SOCS3 expression and a downregulation of the JAK-STAT pathway [41]. Therefore, the suppressive in vivo results by compound A treatment before OVA sensitization might be evoked by the suppression of dendritic cell activation by compound A after the antigen sensitization.

In summary, the present study demonstrated that activation of FFA4, an n-3 PUFA receptor, ameliorates allergic asthma in both antigen sensitization and antigen challenge by suppressing the activation of dendritic cells and mast cells, respectively. Thus, we identified FFA4 as a target of n-3 PUFA action in allergic asthma and suggested that a highly specific agonist of FFA4 could be a therapeutic option for allergic asthma therapy.

## 4. Materials and Methods

### 4.1. Materials

Compound A was purchased from Cayman Chemical (Ann Arbor, MI, USA). Ovalbumin and aluminum hydroxide were obtained from Sigma-Aldrich (St. Louis, MO, USA).

### 4.2. Animals

FFA4 knockout mice (TF0224) were purchased from Lexicon Pharmaceuticals (Woodlands, TX, USA) and backcrossed to BALB/c mice for eight generations [42]. All animals were housed in a laboratory animal facility at Kyung Hee University and provided with food and water *ad libitum*. The mice were housed two per cage in standard plastic cages with sawdust as bedding, with the environments maintained under controlled conditions of temperature ranging between 22–24 °C, humidity level of 60 ± 5%, and alternating light/dark cycles (lights were on between 7:00 h and 19:00 h). The mice were also provided with standard laboratory chow and water. The animal protocol was reviewed by the Institutional Animal Care Committee of Kyung Hee University with respect to the ethics of the procedures used and care (KHSASP-22-102).

### 4.3. Cell Culture

Rat RBL-2H3 mast cells were obtained from the American Type Culture Collection (ATCC, Manassas, VA, USA). RBL-2H3 cells were cultured at 37 °C in a 5% CO_2_-humidified incubator and maintained in 10% (*v*/*v*) heat-inactivated fetal bovine serum containing high-glucose Dulbecco’s modified Eagle medium (DMEM) with 2 mM glutamine, 100 U/mL penicillin, 1 mM sodium pyruvate, and 50 μg/mL streptomycin [43].

### 4.4. Assessment of Degranulation

By measuring the β-hexosaminidase activity in the medium, the degree of degranulation of RBL-2H3 cells was assessed. Monoclonal anti-dinitrophenyl mouse immunoglobulin E and human dinitrophenyl albumin were used to induce degranulation [43].

### 4.5. Asthma Induction in Mice and Administration of Compound A

Following a simple randomization procedure, FFA4-WT and KO 6-week-old female BALB/c mice (22 g) were randomly assigned to one of four treatment groups (*n* = 5): phosphate-buffered saline (PBS)-injected control group, OVA-injected asthma group, compound A treatment before sensitization plus OVA-injected group, and compound A treatment before challenge plus OVA-injected group. Asthma was induced by intraperitoneal injection of 50 μg OVA and 1 mg aluminum hydroxide on D0 and D14 (sensitization). Mice were challenged via exposure to nebulized OVA on D28, D29, and D30 (challenge) [44,45]. Compound A was administered via intraperitoneal injection 30 min before OVA sensitization or challenge. Bronchoalveolar lavage fluids (BALF) were collected from the lungs on D32, and the cell population of BALF cells was analyzed after staining.

### 4.6. Cell Counting and Analysis in BALF

Using a Cellspin^®^ centrifuge (Hanil Electric, Seoul, Korea), immune cells in the BALF were adhered to a glass slide and fixed in methanol for 30 s. Cells were stained with May-Grünwald solution for 8 min, followed by Giemsa solution for 12 min.

### 4.7. Histological Examination of the Lungs

Lung tissue sections were prepared from the mice of each group. Hematoxylin and Eosin (H&E) and periodic acid–Schiff (PAS) staining were conducted to identify mucus-secreting goblet cells and eosinophil infiltration, respectively. Schiff’s reagent was used for PAS staining, and hematoxylin and eosin reagents were used for H&E staining [46].

The degree of lung inflammation was measured by a treatment-blind observer using a subjective scale of 0–3. Mucin-secreting cells stained with PAS in the airways were counted in two lung sections per mouse. At the same time, we also measured the length of the bronchi basal lamina using ImageJ software (National Institute of Health). Mucus production was expressed as the number of PAS-positive cells per millimeter of bronchiole [44].

### 4.8. Quantitative Real-Time PCR

To assess the expression levels of inflammatory markers in the BALF and lungs of mice, first-strand cDNA was first synthesized from total RNA isolated using TRIzol reagent (Invitrogen, Waltham, MA, USA); total RNA was isolated from the BALF and lung tissues and transcribed to cDNA with Moloney Murine Leukemia Virus Reverse Transcriptase (Promega, Madison, WI, USA). Quantitative PCR was performed using Thunderbird Next SYBR qPCR Mix (Toyobo, Osaka, Japan) and CFX Connect Real-Time system (Bio-Rad, Hercules, CA, USA). Thermal-cycling conditions were as follows: one cycle at 95 °C for 4 min, forty cycles at 95 °C for 30 s and 57 °C for 30 s, one cycle at 95 °C for 30 s. The expression of individual genes was normalized to the levels of GAPDH.

### 4.9. Statistical Analysis

Results are expressed as means ± standard deviation (SD) or standard error of the mean (SEM). Statistical significance of analysis of variance (ANOVA) was performed, followed by Turkey’s post hoc test using GraphPad Prism software (GraphPad Software, Inc., La Jolla, CA, USA). Statistical significance was set at *p* values < 0.05.

## Data Availability

The study does not report any data.
